# Is it the time to implement the routine use of distress thermometer among Egyptian patients with newly diagnosed cancer?

**DOI:** 10.1186/s12885-020-07451-7

**Published:** 2020-10-27

**Authors:** Nashwa Abd El-Aziz, Salah Khallaf, Waleed Abozaid, Ghada Elgohary, Ola Abd El-Fattah, Mai Alhawari, Safaa Khaled, Azza AbdelHaffez, Ehab Kamel, Sherif Mohamed

**Affiliations:** 1 Department of Medical Oncology, South Egypt Cancer Institute, Assiut University, Assiut, Egypt; 2 Hematology-Oncology Department, King Khaled University hospital, King Saud University, Riyadh, Kingdom of Saudi Arabia; 3 Department of Clinical Oncology, Faculty of Medicine, Mansura University, Mansura, Egypt; 4 Department of Adult Hematology/Internal Medicine, Ain Shams University, College of Medicine, Cairo, Egypt; 5 Department of Clinical Oncology, Faculty of medicine, Assiut University, Assiut, Egypt; 6 Department of Internal Medicine, Clinical Hematology Unit/Unit of Bone Marrow Transplantation, South Egypt Cancer Institute, Assiut University, Assiut, Egypt; 7 Department of Medical Physiology, Faculty of Medicine, Assiut University, Assiut, Egypt; 8 Department of Pyschiatry, Faculty of Medicine, Assiut University, Assiut, Egypt; 9 Department of Chest Diseases and Tuberculosis, Faculty of Medicine, Assiut University, Assiut, Egypt

**Keywords:** Cancer, Distress thermometer, Egypt, New, Screening

## Abstract

**Background:**

The distress thermometer (DT) is an effective tool for identifying distress among cancer patients worldwide. However, DT has not been studied in Egyptian patients. We aimed to study the prevalence of distress among Egyptian patients with different types of cancers using DT.

**Methods:**

A total of 550 patients with newly diagnosed hematological and solid cancers who were followed up at 3 Oncology Centers in Egypt were enrolled. They completed a sociodemographic and clinical status questionnaire, the DT and the Problem List (PL) scale.

**Results:**

At a DT cut-off score of ≥4, 46% of patients had significant distress, which was related to the tumor site and stage. The most frequent problems reported were treatment decision (64.4%), worry (47%), and fears (44.5%). In univariate logistic regression analysis, participants who had significant distress described 23 out of 36 problems in the practical, family, emotional, and physical areas. After adjustment to sociodemographic and clinical characteristics, multivariable analysis confirmed that insurance, depression, fear, sadness, worry, loss of interest in usual activity, and sleep were independent factors associated with significant distress in cancer patients.

**Conclusions:**

Almost half of Egyptian patients newly diagnosed with cancer reported significant distress. Those who had significant distress described extra problems in the practical, family, emotional, and physical areas. We recommend the routine use of DT for screening Egyptian patients with cancer, as well as the involvement of the psycho-oncology and social services, at the time of their initial diagnosis.

## Background

Cancer is a leading cause of morbidity and mortality worldwide. In a recent study from Egypt [1], age-standardized incidence rates per 100,000 for cancer were 175.9, 157.0, and 166.6 for males, females, and both sexes, respectively. The commonest sites for cancer were; liver (33.6%) and bladder (10.7%) among men, breast (32.0%) and liver (13.5%) among women, and liver (23.8%), breast (15.4%), and bladder (6.9%) for both sexes, respectively. By 2050, a 3-fold increase in incident cancer relative to 2013 is expected [1]. The National Comprehensive Cancer Network (NCCN) defines distress as an emotionally unpleasant psychological (cognitive, behavioral, emotional), social, and/or spiritual experience that might interfere with a patient’s ability to effectively cope with cancer, its physical symptoms, and its treatment [2]. Unfortunately, distress is quite common among cancer patients, with a reported clinically significant distress to be between 20 and 47% during course of the disease [3]. Negative impacts of high levels of distress on cancer patients include less adherence to treatment plans, dissatisfaction with overall care, poorer quality of life, and even poorer survival rates [4, 5]. Because of these drawbacks, it is thought that systematic screening of patients for distress is the effective way that allows timely support for those who are most in need [6, 7]. Therefore, it is not surprising to find that many international regulatory organizations and professional societies [3, 8, 9], have recommended the routine screening and management of distress as an integral aspect of whole-person cancer care.

Then, the onco-medical team taking care of cancer patients has to carry out screening their patients starting with the selection of a screening tool that is suitable in terms of briefness, precision, and acceptability [7, 8]. Being a quick, easy and valid instrument to recognize, diagnose and provide prompt management of distress in cancer patients, the distress thermometer (DT) represents the ideal screening tool in that regards. Review of the literature had shown that DT has been investigated and validated as an effective screening tool for detecting distress among patients with various types of cancer, such as prostate carcinoma [10], lung cancer [11], and breast cancer [12]. Moreover, the original NCCN English version [13] has been successfully translated into several languages, including Dutch [14], Japanese [15], Turkish [16], Italian, Spanish and Portuguese [17], and recently its Arabic version has been validated in Saudi cancer patients [18].

To the best of our knowledge, the DT has not been studied in Egyptian patients with cancer. Therefore, the aims of this study were to study the prevalence of emotional distress among Egyptian patients with different types of cancers at their initial diagnosis, to evaluate the value of implementing the DT as screening tool in those patients, and to identify the most significant factors of the DT problem list (PL) that account for such distress. Identification of such distress and its subsequent management may improve the patients’ outcomes and quality of life.

## Methods

### Study population

An ethical approval was obtained from the Institutional Review Committee of South Egypt Cancer Institute (No: 483). Egyptian cancer patients who have been newly diagnosed with different types of hematological malignancies and/or solid cancers and consulted one of the three Oncology Centers (South Egypt Cancer Institute (SECI), Assiut University Hospital (AUH), and Mansura University Hospital (MUH) were enrolled into the study. Also, the study enrolled some referred lung cancer patients to the Oncology Department of either AUH or SECI. Eligible patients were Egyptian adults (≥ 18 years old), of both genders, who had confirmed diagnosis of cancer (either hematological or solid malignancy), with an adequate command of speaking and reading the Arabic language, with an Eastern Cooperative Oncology Group (ECOG) performance status of < 3, and gave an informed consent. Patients having history of, or undergoing treatment for psychiatric illness were excluded.

### Sociodemographic data and medical status

The patients’ sociodemographic and clinical features were retrieved from the patients’ medical records. Standard sociodemographic data were collected including age, marital status, education level, employment status, performance status, type and stage of cancer as defined by the TNM classification system, and the type of treatment will be received.

### Distress thermometer (DT)

The recently validated Arabic version of DT [18] was used for screening patients of the current study for distress with a cut-off score of ≥4 for significant distress. The study objectives and procedure were fully explained to the eligible patients. Screening was carried out for newly diagnosed patients with cancer at their first out-patient visit or in-patient admission. Patients were asked to rate their distress in the past week on an 11-point visual analog scale ranging from 0 (no distress) to 10 (extreme distress) [18]. Patients were then asked to fill in the Problem List (PL) that accompanies the visual image of the DT to check whether or not (yes/no) they have any of the problems listed during the previous 7 days [2, 18]. For patients who are illiterate, a research assistant helped the patient to rate their distress and fill in the PL. This PL consisted of 36 problems of five grouped categories; spiritual/religious concerns, practical problems, family problems, emotional problems, and physical problems. Correlation between the PL and DT was carried out to identify the nature of the distress and related factors.

### Statistical analysis

The Statistical Package for Social Science; SPSS, version 22 (SPSS Inc., Chicago, IL, USA) has been used for data analysis. The mean score, the standard deviation, the median score and the frequency distribution of the DT have been explored using descriptive statistical analysis. All *P*-values were two-tailed. A P-value *<* 0.05 was considered statistically significant. Binary logistic regression test was carried out to explore the association between the DT cut-off scores of 4 [18] and the demographic and clinical variables, while binary and multivariable logistic regression tests were used to analyse the association between the DT cut-off scores and individual items in the PL.

## Results

### Sociodemographic and clinical characteristics

A total of 550 patients participated in the current study. The median age was 55 (range 18–85) years. Females constituted 60% of the participants. Three hundred thirty-three (79%) patients scored two or less on the ECOG performance status. Different types of solid and hematological malignancies were included; the majority were solid tumors (465/550, 85%) versus 85/550,15% hematological malignancies. Among the solid tumors, the most common types were breast cancer (32.7%), gastrointestinal cancers (23%), and genitourinary (13%). Despite that all patients were newly diagnosed with cancer, 56% of them had stage IV at presentation. Forty-one percent of patients had associated medical comorbidities. Table 1 details the demographic data of the study cohorts.

**Table 1:** Clinical and sociodemographic characteristics of the study subjects (*n* = 550) and their association with the DT score ≥ 4^a^

Characteristic	Overall *N* = 550 (%)	DT cut off ≥ 4 N (%)	DT cut off < 4 N (%)	*P*-value
**Age in years**				
Median (range)	55 (18–85)			
Mean ± SD	51.37 ± 15.29	49.9 ± 14.86	52.62 ± 15.55	< 0.001
**Gender**				< 0.001
Male	224 (40)	82 (37)	142 (63)	
Female	326 (60)	172 (53)	154 (47)	
**Marital Status**				0.803
Single	66 (12)	30 (45)	36 (55)	
Married	393 (72)	178 (45)	215 (55)	
Divorced	16 (3)	8 (50)	8 (50)	
Widow	75 (13)	38 (51)	37 (49)	
**Educational level**				0.013
Non-educated	38 (7)	25 (66)	13 (34)	
Educated	512 (93)	229 (45)	283 (55)	
**Occupation**				0.820
Student	30 (5)	14 (47)	16 (53)	
No job	371 (68)	174 (47)	197 (53)	
Job	149 (27)	66 (44)	83 (56)	
**Monthly Income (EP)**				0.484
< 5000	305 (55)	147 (48)	158 (52)	
5000–10,000	133 (24)	63 (47)	70 (53)	
10,000–15,000	75 (14)	30 (40)	45 (60)	
> 15,000	37 (7)	14 (38)	23 (62)	
**Health Insurance**				0.209
Yes	108 (20)	44 (41)	64 (59)	
No	442 (80)	208 (47)	234 (53)	
**Chronic disease**				0.055
Present	211 (38)	107 (51)	104 (49)	
Absent	339 (62)	147 (43)	192 (57)	
**Tumour Site**				0.006
Head and Neck	17 (3)	10 (59)	7 (41)	
Breast	180 (33)	93 (52)	87 (48)	
Lung	41 (8)	24 (59)	17 (41)	
GIT	127 (23)	56 (44)	71 (56)	
Genitourinary	71 (13)	29 (41)	42 (59)	
Musculoskeletal	29 (5)	6 (21)	23 (79)	
Haematological	85 (15)	36 (42)	49 (58)	
**Type of malignancy**				0.486
Hematological	85 (15)	36 (42)	49 (58)	
Solid tumors	465 (85)	218 (47)	247 (53)	
**Stage**				0.001
Stage 1	29 (5)	6 (21)	23 (79)	
Stage II	108 (19)	20 (19)	88 (81)	
Stage III	109 (20)	24 (22)	85 (78)	
Stage IV	304 (56)	204 (67)	100 (33)	

^a^ *DT* distress thermometer, *EP* Egyptian pound, *GIT* gastrointestinal. For age, data are expressed in mean ± standard deviation and t-test with 95% confidence interval were carried out to compare age between the 2 groups of DT cut-off < 4 and ≥ 4. For other sociodemographic characteristics, data are expressed in numbers and percent and Chi-square tests were used to compare the significance of differences between the 2 groups of DT cut-off < 4 and ≥ 4

### Data from distress thermometer and problem list analysis

Two hundred fifty-four (46.2%) patients had significant distress (DT cut off score ≥ 4). The patients’ average DT score was 3.7. There were significant differences between patients with significant distress (DT cut off score ≥ 4) and those without significant one; with regards to age (*p* < 0.001), gender (*p* < 0.001), educational level (*p* = 0.013), tumor site (*p* = 0.006), and stage of the disease (*p* = 0.001). Table 1 shows these differences. The most frequent problems reported on the practical domain of the PL were, in descending order, treatment decision (64.4%), worry (47%), fears (44.5%), and pain (42.2%). Table 2 shows these details.

**Table 2:** The most frequent problem list items among the studied patients (*n* = 550)

Problems List	No. of patients	%
Treatment decision	354	64.4
Worry	258	47.0
Fears	245	44.5
Pain	232	42.2
Dealing with partner	193	35.1
Sleep	178	32.4
Sadness	168	30.5
Nausea	156	28.4
Eating	156	28.4
Depression	147	26.7
Constipation	141	25.6
Child care	133	24.2
Loss of interest	129	23.4
Sexual	125	22.7
Indigestion	124	22.5

### Association between significant DT score and the PL items

Binary logistic regression showed that DT score of 4 or more was found to have a statistically significant correlation with most of the PL items including; insurance, family health issue, depression, fear, nervousness, sadness, worry, loss of interest in usual activity, religious concerns, appearance, bathing and dressing, breathing, diarrhea, fatigue, feeling swollen, fever, getting around, indigestion, mouth sores, nausea, pain, substance abuse, and sleep. After adjustment to sociodemographic and clinical characteristics, multivariable analysis confirmed that insurance, depression, fear, sadness, worry, loss of interest in usual activity, and sleep, were independent factors associated with significant distress in cancer patients. Table 3 details these associations.

**Table 3:** The association between the Distress Thermometer (DT) score ≥ 4 and the Problems List items of 550 cancer patients

Problem list	Item Present (%)	DT cut-off ≥ 4 N (%)	DT cut-off < 4 N (%)	OR (95% CI)	Adjusted OR (95% CI)
**Child care**	133 (24)	63 (47)	70 (53)	0.9 (0.623–1.362)	
**Housing**	124 (22)	64 (52)	60 (48)	0.7 (0.496–1.106)	
**Health Insurance**	108 (20)	44 (41)	64 (59)	1.3 (0.543–1.866)*	7.6 (0.042–18.029)***
**Transportation**	111 (20)	53 (48)	58 (53)	0.9 (0.598–1.377)	
**Work and school**	36 (6)	20 (56)	16 (44)	1.2 (0.333–1.299)	
**Treatment decisions**	354 (64)	164 (47)	190 (53)	0.9 (0.655–1.340)	
**Dealing with children**	125 (23)	58 (46)	67 (54)	0.9 (0.655–1.477)	
**Dealing with Partner**	194 (35)	99 (51)	95 (49)	0.7 (0.514–1.039)	
**Ability to have children**	87 (16)	43 (49)	44 (51)	0.8 (0.532–1.332)	
**Family Health Issue**	74 (13)	44 (59)	30 (41)	1.4 (0.322–1.171)*	
**Depression**	147 (27)	132 (90)	15 (10)	8.8 (0.027–3.458)***	6.9 (0.067–19.390)***
**Fear**	245 (45)	178 (73)	67 (27)	2.6 (0.082–1.731)***	3.9 (0.134–13.690)***
**Nervousness**	126 (23)	84 (67)	42 (33)	2.0 (0.216–1.696)***	
**Sadness**	168 (31)	136 (81)	32 (19)	4.2 (0.066–2.277)***	2.0 (0.260–7.020)**
**Worry**	258 (47)	178 (69)	80 (31)	2.2 (0.017–1.874)***	4.4 (0.089–14.021)***
**Loss of interest in usual activity**	129 (23)	104 (80)	25 (20)	4.1 (0.081–2.518)***	3.1 (0.157–11.310)***
**Religious**	24 (4)	21 (88)	3 (12)	7.0 (0.033–2.412)***	
**Appearance**	100 (18)	67 (67)	33 (33)	2.0 (0.218–1.525)***	
**Bathing and Dressing**	66 (12)	44 (67)	22 (33)	2.0 (0.219–1.519)***	
**Breathing**	101 (18)	61 (61)	40 (39)	1.5 (0.313–1.163)*	
**Change in urination**	83 (15)	39 (47)	44 (53)	0.8 (0.593–1.511)	
**Constipation**	141 (26)	70 (50)	71 (50)	0.9 (0.554–1.193)	
**Diarrhea**	80 (14)	49 (61)	31 (39)	1.5 (0.304–1.190)*	
**Eating**	156 (28)	87 (56)	69 (44)	1.2 (0.312–1.245)	
**Fatigue**	269 (49)	163 (61)	106 (39)	1.5 (0.299–1.156)*	
**Feeling Swollen**	113 (21)	77 (68)	36 (32)	2.1 (0.201–1.603)***	
**Fever**	71 (13)	50 (70)	21 (30)	2.3 (0.178–1.764)***	
**Getting Around**	91 (17)	62 (68)	29 (32)	2.1 (0.205–1.586)***	
**Indigestion**	125 (23)	75 (60)	50 (40)	1.5 (0.317–1.149)*	
**Memory and Concentration**	98 (18)	54 (55)	44 (45)	1.2 (0.409–0.986)	
**Mouth Sores**	72 (13)	45 (62)	27 (38)	1.7 (0. 275–1.300)**	
**Nausea**	156 (28)	98 (63)	58 (37)	1.7 (0.259–1.351)**	
**Dry Nose &Congestion**	74 (13)	40 (54)	34 (46)	1.1 (0.425–1.142)	
**Pain**	232 (42)	143 (62)	89 (38)	1.6 (0.228–1.477)***	
**Sexual**	125 (23)	72 (57)	53 (43)	1.3 (0.361–0.810)	
**Itching and Dry Skin**	86 (16)	44 (51)	42 (49)	1.0 (0.491–1.231)	
**Tingling sensation in hands and feet**	98 (18)	52 (53)	46 (47)	1.1 (0.453–1.088)	
**Substance Abuse**	35 (6)	31 (89)	4 (11)	9.0 (0.034–3.411)***	
**Sleep**	178 (32)	129 (72)	49 (28)	2.6 (0.127–1.998)***	3.6 (0.155–12.921)***

*OR* Odds Ratio, *CI* confidence interval

* *P*-value < 0.05, ** *p* < 0.01, *** *p* < 0.001

## Discussion

To the best of our knowledge, this is the first study that evaluates the problem of emotional distress among a large number of newly diagnosed patients with different types of cancer, attending three University cancer centers in Egypt.

Using the Arabic version of DT [18] we identified that 46% of our cancer patients had significant distress, which was significantly related to age, gender, educational level, tumor site, advanced stage of the disease, as well as many items of the problem list. This is the second study, after that of Alosaimi, et al [18], that utilizes the Arabic version of the DT in screening a large number of patients with different types of cancer for emotional distress. The NCCN introduced the DT as a screening tool to identify sources of distress at the initial visit soon after diagnosis and at each visit, though the screening schedule may be judged as clinically indicated [2]. The DT is a single-item tool using a zero (no distress) to 10 (extreme distress)–point Likert scale resembling a thermometer. The patient rates his/her level of distress over the past week [2, 9]. The NCCN Problem List for patients is a 39-item supplemental list of potential sources of distress that is incorporated as an essential part of the assessment to assist the provider in identifying distress. As this PL provides a comprehensive list of categories, including practical, family, physical, and emotional problems, as well as spiritual/religious concerns, it covers almost all aspects that might attribute to distress among cancer patients [2, 3, 9, 18]. We think that, despite the apparent simplicity of DT, it covers most-if not all-problems might be faced by any study population (i.e. populations with different racial, religious, social and financial aspects), and worldwide. So, it is not surprising that DT has been successfully translated from English into several languages [13–16, 18].

Almost half of our cohorts had a significant distress. This is in accordance with the worldwide reported prevalence rates of 20 to 52% of cancer patients [2, 9, 19] for distress among cancer patients. Particularly, our figures are very similar to that (45.89%) reported by Hahn, et al [20].

Our study revealed that significant distress is related to age, gender, educational level, and tumor site, and stage of the disease. We enrolled a large number of patients with different types and sites of cancers, which may explain these significant associations. Despite it is thought that all cancer patients are at risk for distress, studies had identified specific risk factors like age and gender that increase the prevalence of distress among certain cancer groups. In our cohort, younger patients experienced higher levels of distress which is in agreement with previous studies [12]. Studies have shown gender differences, with reported higher levels of distress among women [12, 21, 22]. Interestingly, 56% of the current study cohort had stage IV at their initial presentation to the oncologist. Despite that this figure does not reflect advanced stage at presentation for certain tumor type, it gives an alarm about an observation that is seen in low- and middle-income countries (LMCs). In these regions, late-stage presentations can be attributed to several factors including; lack of proper health infrastructure and screening programs, limited access to health-care facilities, lack of community awareness, and social barriers impeding early detection and treatment programs [23, 24]. Interestingly, the prevalence of psychological distress differs in different cancer sites. It was observed that patients diagnosed with cancers of the breast, head and neck, colon, lung, brain, or pancreas experience greater distress [25]. On the other hand, our results contradict previous reports where no significant links between the DT and sociodemographic and clinical characteristics were found [3, 13, 15, 18]. These different findings could be contributed to differences in the study populations, ethnic groups, numbers, and types of cancer among different studies. The most frequent problems reported on the practical domain of the PL were, treatment decision (64.4%), worry (47%), fears (44.5%), and pain (42.2%).

These encountered problems are similar to the ones found by Alosaimai and co-workers [18] which could be explained by the cultural and emotional demographics similarities between the two studied Arabian populations. Even more, these encountered problems are the most challenging to the clinicians in their daily practice. It is though that the relation between dissatisfaction with the treatment decision and the experienced distress is bidirectional [26]. Hence, to alleviate treatment decision related distress, it is preferable to provide the patients with full prognostic information, treatment plans, possible adverse effects and respect their future expectations [27].

The results of this study had highlighted the importance of early screening cancer patients (at their initial diagnosis with cancer) for distress. We have seen that participants who scored 4 or more on the DT described extra problems in the practical, family, emotional, spiritual/religious and physical areas (23 out of 36 problems) than patients who scored below this cut-off score. Although degrees vary, this finding suggests that a wide range of problems contributes to distress in cancer patients [28], which is again consistent with many similar studies performed worldwide, and among various cancer populations [11–14, 18, 28]. Despite that further analysis had identified the presence of only 7 out of 36 problems, as independent factors associated with significant distress in our cohort, still this is considered a large number of problems for a cancer patient to challenge.

These findings are very similar to those observed by Alosaimi, et al [18], while they are quite different from those reported by McFarland, et al [28] who stated that only three factors were significantly associated with distress (breathing problem, eating, and nausea). This agreement with Alosaimi, et al [18] is attributed to the fact that both studies recruited Arabian population with nearly similar cultural, spiritual and emotional factors. Notably, financial and emotional concerns were prominent issues among our cohort. We had observed prominent anxiety depression symptoms (depression, fear, nervousness, sadness and worry). Finding of such problems confirms the importance of implementing “routine” screening of cancer patients for emotional distress in our society. Sleep disturbances are quite common among cancer patients, ranging from 25 to 59% [6, 18, 19]. Consequently, these disturbances may lead to poor quality of life, compliance to treatment and hence the development of depressive stigmata [18, 19].

In comparison, the contrary with McFarland et al [28] could be explained by the differences in patients’ numbers and cancer type between the two studies. We included a wide range of malignancies, while they included only patients with breast cancer.

In essence, barriers to screening for distress exist and they should be taken into consideration while implementing a screening program for distress [29]. Simply, patients may have misunderstanding about what the word “distress” means and to what extent it ranges [30]. Screening through the prior 1 week may be not sufficient for some patients to experience their distress. Patient barriers to screening include cultural differences as well as literacy [31]. Unfortunately, 49% of our study cohorts had low education levels (illiteracy and primary school education). Institutional barriers may include lack of privacy for screening, insufficient time and training, poor documentation of results, and lack of resources for patient referrals [30–32].

Overall, findings of the current study confirm the importance of “routine” screening of cancer patients for emotional distress. Also, they support the importance of early recognition of distress among cancer patients that comes from the possibility of overcoming its hazardous sequelae. These sequelae are difficulty in taking a treatment decision, noncompliance with cancer treatment, frequent unneeded medical visits to caregivers, more pressure on the treating team [4, 9] and more hospitalization [33]. Moreover, in some studies, it has a negative impact on survival [34]. It is well known that screening programs improve patient outcomes only when linked to an effective system of assessment and treatment. Therefore, cancer centres should implement DT screening only after developing a plan for the timely evaluation of distress, reviewing its results and managing patients whose scores suggest clinically significant distress, including making appropriate referrals based on the problem areas specified on the PL [8].

Being the first multicenter study with a large number of patients of different types of cancer does not guarantee that it has no limitations. Possible limitations include possible bias from including many types of cancers and possible convenience sampling which may affect the generalizability of the study findings to all cancer patients in Egypt.

## Conclusion

The study found that almost half of Egyptian newly diagnosed cancer patients reported significant distress. This distress was significantly related to age, gender and stage of cancer at presentation. Patients who scored 4 or more on the DT described additional problems in the practical, family, emotional, and physical areas than patients who scored below this cut-off score. We recommend the routine use of DT for screening Egyptian patients with cancer, as well as the involvement of the psycho-oncology and social services at the time of initial diagnosis. Further multicenter studies are warranted to explore this phenomenon.

## Data Availability

The datasets used and/or analyzed during the current study are available from the corresponding author on reasonable request.

## Abbreviations

DT: Distress thermometer
ECOG: Eastern Cooperative Oncology Group
LMCs: Low- and middle-income countries
NCCN: National Comprehensive Cancer Network
PL: Problem list
TNM: Tumor-node-metastasis
